# Predictors of immobility following surgery for periprosthetic hip fractures

**DOI:** 10.1007/s00402-025-06145-8

**Published:** 2025-12-02

**Authors:** Christopher Lampert, Florian Pachmann, Kathrin Pfahl, Boris Michael Holzapfel, Wolfgang Böcker, Tobias Helfen, Carl Neuerburg, Leon Faust

**Affiliations:** Department of Orthopaedics and Trauma Surgery, Musculoskeletal University Center Munich (MUM), LMU Klinikum, Munich, Germany

**Keywords:** Periprosthetic femoral fracture, Weight-bearing protocol, Postoperative mobility, Orthogeriatric

## Abstract

**Background:**

Periprosthetic femoral fractures (PPF) following total hip arthroplasty (THA) are a growing clinical challenge, particularly in the elderly. Postoperative mobility is a key determinant of outcome, yet little is known about how surgical treatment and weight-bearing protocols influence functional recovery in this population.

**Methods:**

We conducted a retrospective cohort study of 186 patients aged ≥ 65 years treated surgically for Vancouver type B or C PPF between January 2017 and August 2023 at a level 1 trauma center. Patients underwent either open reduction and internal fixation (ORIF) or revision arthroplasty. Postoperative weight-bearing protocols were categorized as full weight bearing (FWB) or partial weight bearing (PWB). The primary outcome was mobility status at hospital discharge. Binary logistic regression was used to identify independent risk factors for immobility.

**Results:**

Of the 186 patients included (mean age 82.4 ± 7.4 years), 64.5% were mobilized with FWB. In the ORIF group, patients allowed FWB were significantly older (85.5 ± 7.4 vs. 80.2 ± 6.3 years, *p* < 0.001). No significant differences in mobility, complication rates, revision surgery, or in-hospital mortality were observed between weight-bearing regimens, regardless of the type of surgery. Logistic regression identified intraoperative blood transfusion (OR = 3.17) and higher ASA scores (ASA III: OR = 9.10; ASA IV: OR = 32.03) as independent predictors of postoperative immobility. Neither the postoperative weight-bearing protocol nor the surgical procedure had a significant impact on mobility outcomes.

**Conclusion:**

This study examines postoperative weight-bearing strategies on clinical outcomes in patients with periprosthetic hip fractures and simultaneously identifies independent risk factors associated with postoperative immobility. Our findings suggest that mobility outcomes are more influenced by patient-related factors than by surgical procedure. Our findings support the safe implementation of full weight-bearing protocols in elderly patients following periprosthetic femoral fractures, regardless of the type of surgical intervention.

## Introduction

The number of total hip arthroplasty (THA) continues to grow annualy [21]. In addition to ageing patients with osteoporosis, increasingly younger and more active patients are also receiving joint replacements [25]. Together with the increasing number of primary arthroplasties, this leads to an increased incidence of periprosthetic femoral fractures (PPF). PPF rank among the most common causes of revision surgery after THA [6]. They are associated with high morbidity, increased short- and long-term mortality, and a substantial decline in postoperative mobility [15].

Treatment decisions for PPF are commonly guided by the Vancouver classification system, which considers fracture location, implant stability, and bone quality. When the prosthesis remains stable, internal fixation using locking plates or intramedullary nails is generally indicated. However, in the presence of a loose femoral component, as seen in Vancouver type B2 or B3 fractures, revision arthroplasty is typically required [2].

Postoperative mobility is a critical determinant of clinical outcome, especially in elderly, orthogeriatric patients. Evidence from hip fracture patients demonstrates that fewer than 50% of patients regain their pre-fracture mobility status, with many remaining dependent on long-term care [27, 29]. Reduced mobility not only impairs quality of life but is also strongly associated with increased mortality [24]. Early full weight-bearing mobilization plays a pivotal role in postoperative recovery, especially in patients after hip fracture surgery [26]. On the other hand weight-bearing restrictions have been shown to negatively affect mobility and functional outcomes [22

The aim of this study is to analyze the effect of postoperative weight bearing restrictions on clinical outcomes following periprosthetic femoral fractures. Given the increasing prevalence of PPF and the vital role of mobility in recovery and survival, we further aimed to identify independent risk factors for postoperative immobility following periprosthetic hip fracture surgery.

## Materials and methods

### Patients

This study was conducted as a retrospective cohort study. Patients diagnosed with a PPF between January 2017 and August 2023 at a level 1 trauma center were screened for inclusion. Fractures were classified using the common Vancouver classification [2]. Primary inclusion criteria for participants were Vancouver type B1, B2, B3 and C fractures, which have been treated surgically with open reduction and internal fixation (ORIF) or revision arthroplasty. The Vancouver classification was determined based on intraoperative findings. Minimum age for inclusion was 65 years. Patients with additional fractures or known immobility pre-fracture due to comorbidities were excluded from the analysis. All patients received standardized analgesic therapy based on the WHO analgesic ladder. The study was conducted in accordance with the Declaration of Helsinki. Ethical approval was given by the local ethics committee (Ref.-No. 25–0704).

### Outcome parameters

Patient data were retrieved anonymously from the digital hospital database. Demographic and physical data including age, gender and body-mass-index (BMI) were acquired. Overall physical status was assessed using the American Society of Anesthesiologists (ASA) classification. Detailed data regarding surgery were collected including type of surgery, duration of surgery and intraoperative blood transfusion protocol. Temporary postoperative care and treatment on an intensive care or intermediate care unit was captured. Postoperative complications such as pneumonia, surgical revision, deep vein thrombosis (DVT), pulmonary embolism (PE), urinary tract infection (UTI) and wound complications were investigated. All patients received physiotherapy from the first postoperative day on. Regular documentation of postoperative mobility status was present in all patients` data records. The documentation of postoperative mobilization and weight-bearing pattern (full weight-bearing as tolerated (FWB) vs. partial weight-bearing (PWB)) was analyzed. Patients who were bedridden, sitting or standing were classified as immobile. All patients able to walk with or without a walking aid were considered mobile. Mobility status upon discharge was the primary outcome parameter of this study.

### Statistical analysis

Descriptive statistics on demographic data and patient characteristics were obtained for the whole cohort and both groups separately. Depending on the results of the Shapiro-Wilk-Test, means of normally distributed data were compared with Student’s t-Test. Means of non-normally distributed data were compared with the Mann-Whitney-U-Test. Association of categorial variables was analyzed with Chi-Square test, Bonferroni adjustment was performed as post-hoc test. All parameters and values stated were complete. Predictors for immobility by the end of the inpatient stay were evaluated using a binary logistic regression. Prevalence of immobility was defined as dichotomous dependent variable. Model significance was tested with Chi-Square-Test, effect size was calculated with Cohen’s f^2^. An odds ratio ≥1 indicated a higher chance of immobility. The level of significance was set at *p* < 0.05. The analyses were performed with IBM SPSS Statistics Version 29 (IBM Germany GmbH, Ehingen, Germany).

## Results

A total of 186 patients was included in this study. 62.9% of all patients were female (*n* = 117). The mean age was 82.4 ± 7.4 years (range 65–100 years). Detailed descriptive data on the ASA score, fracture classification, respective type of surgery, and respective postoperative mobilization scheme can be seen in Table 1. Most patients were classified as ASA III (65.6%), followed by ASA II (24.2%). The most common fracture type was Vancouver B2 (39.2%), followed by B1 (26.3%), B3 (22.6%), and C (11.8%). The majority of B1 fractures were treated with open reduction and internal fixation (ORIF) (95.9%), whereas B2 and B3 fractures were predominantly treated with revision hip arthroplasty (76.7% and 90.5%, respectively). Vancouver C fractures were treated with ORIF in 90.2% of cases. Among patients treated with ORIF, 63.6% were mobilized with full weight bearing (FWB), while 36.4% were restricted to partial weight bearing (PWB). Similarly, in the revision arthroplasty group, 65.3% were mobilized FWB and 34.7% PWB.

**Table 1:** Demographic and treatment characteristics of patients with periprosthetic hip fractures

Mean ± SD			
Age (years)	82.4 ± 7.4		
n (%)			
Sex (female)	117 (62.9%)		
ASA			
II	45 (24.2%)		
III	122 (65.6%)		
IV	18 (9.7%)		
V	1 (0.5%)		
Fracture Type (Vancouver Classification)		Surgical treatment	
		ORI	Revision arthroplasty
Total	186 (100%)	88 (47.3%)	98 (52.7%)
B1	49 (26.3%)	47 (95.9%)	2 (4.1%)
B2	73 (39.2%)	17 (23.3%)	56 (76.7%)
B3	42 (22.6%)	4 (9.5%)	38 (90.5%)
C	22 (11.8%)	20 (90.2%)	2 (9.8%)
Postoperative Mobility			
PWB	66 (35.5%)	32 (36.4%)	34 (34.7%)
FWB	120 (64.5%)	56 (63.6%)	64 (65.3%)

*ASA* American Society of anesthesiologists, *ORIF* open reduction internal fixation, *PWB* partial weight bearing, *FWB* full weight bearing, *SD* standard deviation. Values are presented as mean ± standard deviation or percentage unless otherwise indicated.

The first subanalysis investigated differences in the postoperative mobility scheme between partial and full weight bearing for each type of surgical treatment (ORIF and revision arthroplasty). The results for the ORIF group can be seen in Table 2 below. Patients in the FWB group were significantly older than those in the PWB group (85.5 ± 7.4 vs. 80.2 ± 6.3 years, *p* < 0.001). No statistically significant differences were observed between groups in terms of sex distribution, BMI, ASA score. Postoperative complications and rates of surgical revision were low and comparable between the groups. Similarly, there were no significant differences in mobility and discharge disposition, with most patients being discharged to rehabilitation clinics. The mean length of hospital stay did not differ significantly between the two groups (14.5 ± 7.2 vs. 17.1 ± 14.2 days, *p* = 0.495). In-hospital mortality was slightly higher in the PWB group (12.5% vs. 8.9%), but this difference was not statistically significant (*p* = 0.595).

**Table 2:** Comparison of postoperative Weight-Bearing groups following ORIF for periprosthetic hip fractures

ORIF	PWB (*n* = 32)	FWB (*n* = 56)	*p*-value
Age	80.2 ± 6.3	85.5 ± 7.4	**< 0.001**
Sex (female)	24 (75%)	37 (66.1%)	0.382
BMI	25.1 ± 5.1	23.3 ± 4.1	0.088
ASA Score	3 (IQR 0.75)	3 (IQR 0)	0.761
Revision surgery	3 (9.4%)	5 (8.9%)	0.944
Non-surgical complications			
Pneumonia	1 (3.1%)	0	0.183
DVT	0	0	
LAE	1 (3.1%)	0	0.183
UTI	2 (6.3%)	6 (10.7%)	0.483
Impaired wound healing	1 (3.1%)	0	0.183
Discharge to			
Rehabilitation clinic	14 (43.8%)	27 (48.2%)	0.686
Patient’s Home	12 (37.5%)	18 (32.1%)	0.610
Nursing Home	1 (3.1%)	4 (7.1%)	0.434
Hospital transfer	1 (3.1%)	2 (3.6%)	0.911
Immobility	11 (34.4%)	18 (32,1%)	0.830
Length of stay (days)	14.5 ± 7.2	17.1 ± 14.2	0.495
In-hospital Mortality	4 (12.5%)	5 (8.9%)	0.595

*ORIF*, open reduction and internal fixation, *PWB* partial weight bearing, *FWB* full weight bearing, *BMI* body mass index, *ASA* American society of anesthesiologists, *DVT* deep vein thrombosis, *LAE* pulmonary artery embolism, *UTI* urinary tract infection. Values are presented as total numbers with percentages, interquartile range (IQR), or mean ± standard deviation, unless otherwise indicated. Statistically significant *p*-values are shown in bold.

Table 3 shows the comparison of FWB vs. PWB in the revision arthroplasty group. No significant differences were found between groups in demographic data. Surgical revision, complication rates, length of hospital stay and mortality rates were comparable between the groups. A non-significant trend toward higher immobility in the FWB group was observed (32.8% vs. 17.7%, *p* = 0.053). Discharge destination also did not differ significantly; however, a higher proportion of PWB patients were discharged to their homes (44.2% vs. 25.0%, *p* = 0.053), while nursing home discharges were more frequent in the FWB group (15.6% vs. 5.9%, *p* = 0.161).

**Table 3:** Comparison of postoperative Weight-Bearing groups following revision hip arthroplasty for periprosthetic hip fractures

Revision Arthroplasty	PWB (*n* = 34)	FWB (*n* = 64)	*p*-value
Age	80.3 ± 7.4	81.9 ± 7.2	0.282
Sex (female)	20 (58.8%)	36 (56.3%)	0.805
BMI	25.8 ± 3.8	24.4 ± 3.9	0.114
ASA score	3 (IQR 1)	3 (IQR 0)	0.177
Revision surgery	6 (17.6%)	15 (23.4%)	0.506
Non-surgical complications			
Pneumonia	0	4 (6.3%)	0.137
DVT	0	1 (1.6%)	0.464
LAE	2 (5.9%)	2 (3.1%)	0.511
UTI	6 (17.6%)	9 (14.1%)	0.639
Impaired wound healing	1 (2.9%)	3 (4.7%)	0.677
Discharge to			
Rehabilitation clinic	15 (44.1%)	31 (48.4%)	0.683
Patient’s Home	15 (44.2%)	16 (25%)	0.053
Nursing Home	2 (5.9%)	10 (15.6%)	0.161
Hospital transfer	2 (5.9%)	3 (4.7%)	0.789
Immobility	5 (17.7%)	21 (32.8%)	0.053
Length of stay (days)	17.8 ± 6.8	17.6 ± 9.0	0.515
In-hospital mortality	0	4 (6.3%)	0.137

*ORIF* open reduction and internal fixation, *PWB* partial weight bearing, *FWB* full weight bearing, *BMI* body mass index, *ASA* American Society of Anesthesiologists, *DVT* deep vein thrombosis, *LAE* pulmonary artery embolism, *UTI* urinary tract infection. Values are presented as total numbers with percentages, interquartile range (IQR), or mean ± standard deviation, unless otherwise indicated. Statistically significant *p*-values are shown in bold.

A binary logistic regression model was used to identify factors associated with immobility at the end of the inpatient stay (Table 4). The analysis revealed that intraoperative blood transfusion was significantly associated with increased odds of immobility (OR = 3.17, 95% CI: 1.24–8.10, *p* = 0.016). Similarly, higher ASA scores led to an increased risk of immobility (ASA 3: OR = 9.10, 95% CI: 1.74–47.50, *p* = 0.009; ASA 4: OR = 32.03, 95% CI: 4.43–231.51, *p* < 0.001). Additionally, urinary tract infection (UTI) was significantly associated with a better mobility at discharge (OR = 0.24, 95% CI: 0.06–0.97, *p* = 0.046). Other variables, including age, sex, BMI, type of surgery, surgical duration, and ICU/IMC stay, were not significantly associated with immobility. Especially the postoperative mobility scheme (FWB/PWB) did not significantly increase the risk of postoperative immobility.

**Table 4:** Binary logistic regression analysis of factors associated with immobility at the end of inpatient stay

	OR	95% CI	*p*-value
Age	1.03	0,971-1,093	0.328
Sex (female)	0.598	0,247-1,446	0.253
BMI	0.964	0,871-1,067	0.480
ASA 3	9.096	1,742 − 47,499	**0.009**
ASA 4	32.025	4,43–231,51	**< 0.001**
Type of surgery (ref. Revision arthroplasty)	0.995	0,398-2,484	0.991
Duration of surgery (min)	0.993	0,985-1,001	0.084
Intraoperative blood transfusion	3.173	1,243-8,101	**0.016**
Postoperative Care – IMC	1.634	0,583-4,578	0.351
Postoperative Care - ICU	2.016	0,734-5,538	0.174
Revision surgery	2.7	0,821-8,878	0.102
Pneumonia	2.277	0,32 − 16,187	0.411
PE	10.635	0,599 − 188,847	0.107
Impaired wound healing	1.899	0,186 − 19,349	0.588
UTI	0.243	0,061 − 0,972	**0.046**
Postoperative mobilization (ref. FWB)	1.152	0,485-2,739	0.749

*OR* odds ratio, *CI* confidence interval, *ASA* American society of anesthesiologists, *BMI* body mass index, *IMC* intermediate care unit, *ICU* intensive care unit, *UTI* urinary tract infection, *PE* pulmonary embolism. Significant p-values are in bold.

## Discussion

The management of periprosthetic femoral fractures (PPF) following total hip arthroplasty represent a major challenge in everyday clinical practice that orthopedic surgeons are expected to encounter with increasing frequency in the coming years. Early postoperative mobilization remains a critical determinant of patient outcomes, particularly in geriatric and multimorbid populations. The present study aimed to evaluate the impact of postoperative weight-bearing regimens on clinical outcomes and to identify risk factors associated with postoperative immobility.

Key findings of this study revealed intraoperative blood transfusion and higher ASA scores as significant risk factors for postoperative immobility, while urinary tract infections (UTI) prevented immobility. Notably, neither the type of surgery nor the postoperative weight-bearing protocol had a significant effect on mobility outcomes during the hospital stay. The association between allogenic blood transfusions and adverse outcomes has been previously described in the context of primary hip and knee arthroplasty. Blood transfusions have been linked to an increased risk of surgical site infections as well as elevated 30-day mortality rates [8, 14]. There is evidence that blood transfusions can also predispose to postoperative complications in patients undergoing surgical treatment for periprosthetic fractures [9, 23]. Together with our findings these results underscore the need for careful intraoperative blood management to minimize blood transfusions in the perioperative setting. Accordingly, the use of tranexamic acid reduces intraoperative blood loss and transfusion rates during revision arthroplasty surgery [10, 31]. Higher ASA scores reflect increased comorbidity burden and reduced physiological reserve, which are known predictors of worse functional outcomes and mortality after hip fracture or arthroplasty [19]. This underlines the vulnerability of geriatric patients. Interestingly non-surgical complications like pneumonia or pulmonary embolism showed no effect on postoperative mobility in our study. In contrast, non-surgical complications following hip fracture surgery are known to have a significant impact on patient outcomes, including increased mortality, higher rates of readmission, and prolonged hospital stays [5, 18]. UTI are the most common hospital-acquired infection in patients undergoing surgery for hip fractures [11]. UTI are recognized as a significant contributor to the development of delirium and are associated with extended hospital stays and an increase in patient mortality [5]. A major risk factor for postoperative UTIs is the use of urinary catheters. To minimize the risk of infection, current guidelines recommend removing such catheters within 24 h of placement whenever possible [28].

The choice of surgical treatment for periprosthetic femoral fractures after total hip arthroplasty largely depends on the fracture configuration and the stability of the existing implant. Patients are typically treated with either open reduction and internal fixation (ORIF) or revision arthroplasty. Vancouver B2 and B3 fractures, which are associated with a loose femoral stem, often require revision arthroplasty and are generally linked to worse clinical outcomes [1]. Several studies have reported that osteosynthesis is associated with shorter operative time, reduced need for blood transfusion, fewer overall complications, lower rates of reoperation, and shorter hospital stays when compared to revision arthroplasty [16].

However, functional outcomes appear to be similar between the two approaches. A meta-analysis by González-Martín et al. found only four studies that included a pre- and postoperative functional assessment, yet no significant difference in Parker Mobility Scores was observed between patients treated with ORIF and those treated with revision arthroplasty [7]. These findings align with the results of our study, in which no statistically significant differences in mobility were detected between the two surgical groups. It is important to evaluate postoperative function, such as mobility or quality of life as a primary outcome rather than focusing solely on radiographic healing or readmission rates. Functional recovery is particularly relevant in geriatric patients, where the ability to regain pre-fracture mobility often determines long-term independence and survival.

Two-thirds of patients were mobilized with full weight bearing (FWB), while one-third were restricted to partial weight bearing (PWB), regardless of the surgical procedure performed. Among patients treated with ORIF, those mobilized with FWB were significantly older than those in the PWB group (85.5 ± 7.4 vs. 80.2 ± 6.3 years, *p* < 0.001). In patients undergoing revision arthroplasty, demographic characteristics were comparable between weight-bearing groups. Interestingly, there were no significant differences in mobility, non surgical complications, need for revision surgery, length of hospital stay, or mortality between the two mobilization regimes for both types of surgery.

Similar findings were reported in an analysis of the ACS-NSQIP database, which showed that only 64% of patients undergoing hip fracture surgery were advised to mobilize with full weight-bearing as tolerated [20]. Other studies reflect this pattern of postoperative weight-bearing restrictions. For example, Wu et al. reported that 23% of patients were prescribed limited weight-bearing following hip fracture surgery [30]. To date, there are no specific data available regarding postoperative weight-bearing protocols in the treatment of periprosthetic fractures. However, especially in geriatric populations, the primary goal of fracture management should be to enable early mobilization to prevent complications associated with delayed mobility, such as pneumonia, urinary tract infection, thromboembolism, delirium, and prolonged hospital stay [12, 17]. Although international guidelines uniformly recommend early mobilization beginning on postoperative day one, a considerable proportion of surgeons continue to prescribe restricted weight-bearing [20]. The rationale includes concerns about fixation failure due to poor bone quality, specific fracture patterns, or the chosen method of fixation [4, 22]. In many cases, these decisions are based more on personal clinical experience than on strong evidence [4]. The results of our study support the safe implementation of full weight bearing, as there are no short-term differences in complication, revision, or mortality rates. Interestingly, studies have also shown that elderly patients frequently fail to adhere to partial weight-bearing instructions [3, 13]. This is reflected by the finding that patients in the ORIF group who were mobilized with weight bearing as tolerated were significantly older. This poor adherence to weight bearing regimes suggests that imposed restrictions may not only be ineffective but further reduce functional mobility in this vulnerable population [22]. Further, this may indicate that patients prescribed partial weight-bearing in the present study, were unable to maintain partial weight-bearing. Hence, it is possible that the real load on the lower extremitites did not differ significantly between the PWB and FWB group, which might mask differences in the measured outcomes.

This study has several limitations. First, the retrospective design inherently introduces the risk of selection bias and limits the ability to establish causal relationships. Although weight-bearing protocols were standardized within the institution, the decision to prescribe full or partial weight bearing was ultimately made at the discretion of the treating surgeon, which may reflect unmeasured clinical judgment or patient-specific factors. Second, functional outcomes were assessed based on mobility status at discharge, without follow-up data on long-term functional recovery or return to baseline mobility levels. Furthermore no specific frailty scores were collected.

## Conclusion

There is limited evidence regarding mobility outcomes and the impact of postoperative weight-bearing protocols on clinical outcomes in patients with periprosthetic femoral fractures of the hip. In this study, the type of surgical procedure (ORIF vs. revision arthroplasty) did not significantly influence short-term mobility. Instead, immobility was primarily associated with patient-related factors such as intraoperative blood transfusion and higher ASA scores. Our findings support the safe application of full weight-bearing protocols in elderly patients with periprosthetic femoral fractures. Considering the poor compliance of older patients with partial weight-bearing restrictions and the absence of adverse effects in our cohort, the use of postoperative weight-bearing limitations in this setting should be critically re-evaluated. Future prospective, multicenter studies with long-term follow-up are needed to validate these results and establish evidence-based mobilization strategies for this growing and fragile patient group.

## Data Availability

The datasets generated and analyzed during the current study are not publicly available due to patient privacy and institutional data protection regulations but are available from the corresponding author on reasonable request.
